# Photobiomodulation and Wearable Light Therapies: A Bibliometric Analysis of the Scientific Literature (1970–2025)

**DOI:** 10.3390/ijerph23050610

**Published:** 2026-05-05

**Authors:** Alberto Grossi, Francesca Campoli, Giuseppe Messina, Giuseppe Caminiti, Matteo Vitarelli, Gabriele Morganti, Elvira Padua, Bruno Ruscello

**Affiliations:** 1 Department of Neurosciences, Biomedicine and Movement, University of Verona, 37124 Verona, Italy; matteo.vitarelli@univr.it; 2 Department of Human Science and Promotion of Quality of Life, San Raffaele University, 00166 Rome, Italy; francesca.campoli@uniroma5.it (F.C.); giuseppe.caminiti@sanraffaele.it (G.C.); gabriele.morganti@uniroma5.it (G.M.); elvira.padua@uniroma5.it (E.P.); bruno.ruscello@uniroma5.it (B.R.); 3 Department of Sports Engineering Lab, Tor Vergata University, 00133 Rome, Italy; 4 Cardiology Rehabilitation Unit, Scientific Institute for Research, Hospitalization and Healthcare (IRCCS) San Raffaele, 00163 Rome, Italy; 5 Department of Industrial Engineering, Faculty of Engineering, Tor Vergata University, 00133 Rome, Italy; 6 LUISS SportLab, Luiss Guido Carli University (LUISS), 00197 Rome, Italy

**Keywords:** heliotherapy, laser development, Low-Level Laser Therapy (LLLT), photobiomodulation (PBM), light-based therapies, wearable Light-based medical devices

## Abstract

**Highlights:**

**Public health relevance—How does this work relate to a public health issue?**

- Technological progress, advances in materials science, and medical device manufacturing techniques, together with scientific evidence on the therapeutic use of light, are increasingly making photomedicine a reality for modern therapeutic applications.
- The potential application of photobiomodulation (PBM) through wearable photomedical devices can have a direct impact on reducing public healthcare costs, improving access to care, and ensuring therapeutic continuity by extending treatments to home settings.

**Public health significance—Why is this work of significance to public health?**

- Most therapies used to treat chronic degenerative diseases are invasive and require outpatient treatments. Furthermore, to enhance therapeutic effects, these are often combined with pharmacological therapies, representing an additional long-term health risk.
- A non-invasive, non-pharmacological therapy, with the possibility of integration into smart systems such as PBM, may represent a key solution to overcome these limitations.

**Public health implications—What are the key implications or messages for practitioners, policy makers and/or researchers in public health?**

- Technological and scientific advances in photomedicine are facilitating the development of wearable devices for light-based therapeutic applications.
- PBM represents a non-invasive, non-pharmacological strategy with potential positive impact on the management of chronic diseases.

**Abstract:**

Background: This study aims to map the temporal evolution of light-based therapies and identify emerging technological trends in wearable photobiomodulation (PBM) devices. Materials and Methods: A bibliometric analysis (1970–2025) was conducted using three major databases: Scopus, PubMed, and Web of Science. The initial dataset, consisting of 117 articles, was processed using the Bibliometrix package in R (version 4.5.0), resulting in a final set of 110 articles. The analysis followed the TALL model (Tracking, Analysis, Layout, and Learning). Results: Scientific production on phototherapeutic devices began in the early 2000s, peaking in 2024, showing a productivity pattern typical of emerging or highly specialized fields. The period 2010–2023 represents a central thematic hub in research. During this time, new light sources (OLED and QLED) enabled the development of flexible, wearable, and implantable photonic devices. In the recent period (2024–2025), light-based therapies are increasingly integrated with network-connected biosensing systems for tissues or accessories, allowing adaptive treatments and remote monitoring. However, these next-generation devices are still undergoing consolidation and scientific maturation. Conclusions: The results highlight the rapid evolution of research on light-based therapies toward more integrated and clinically validated approaches, indicating growing scientific interest in personalized wearable PBM devices.

## 1. Introduction

### 1.1. The Evolution of History: From Heliotherapy to Laser Technology

Exposure to sunlight, referred to as “heliotherapy”, has long attracted scientific interest, with early expectations of light as a therapeutic tool. The pioneering work of modern medical science, culminating in the 1903 Nobel Prize in Physiology and Medicine, is attributed to Niels Ryberg Finsen, who used red and blue (non-solar) light to treat various human disorders, particularly Lupus Vulgaris [1]. These insights resonated widely among health advocates and innovators in technology and wellness. John Harvey Kellogg, recognizing the potential healing effects of electric light, developed the “Light Cabinet” or “Light Bath” at the Battle Creek Sanitarium in Michigan [2]. Concurrently, there was a return to heliotherapy, but with a novelty characterized by exposure to sunlight at high altitudes with cold alpine air. Bernhard, who achieved success with non-healing infected wounds, and Rollier, who treated articular tuberculosis as an alternative to traditional surgery, were key figures in this revival [3]. Rollier’s Sun Therapy, which introduced standardized protocols, was widely accepted throughout Europe. With the advent of modern medicine, these treatments lost scientific attention, leading to a “dark mini-era” for light therapy.

### 1.2. The Evolution of History: From Laser Technology to Low-Level Laser Therapy (LLLT)

The second milestone in therapeutic light use began with Theodore H. Maiman and the invention of laser technology in the early 1960s [4]. This initiated a “laser rush”, leading to the development of various laser systems, now widely used across scientific and technological fields [5]. Leon Goldman, considered the father of laser medicine, demonstrated the applicability of this technology in dermatology for tattoo removal and tissue ablation [6,7]. Researchers aimed to better understand the biological effects of laser therapy before clinical application. Paul McGuff studied tumoricidal effects of ruby laser therapy, suggesting that its mechanism was not solely thermal [8]. Mester was attempting to replicate the experiment first conducted by McGuff in Boston, although with inconsistent and non-systematic results. Because Mester’s customized ruby laser possessed only a small fraction of the power of the device used by McGuff, he was unable to reproduce the experiments. Instead, he observed increased hair growth and improved wound healing in rats in which malignant tumors had been surgically implanted [9,10]. This provided the first indication that low-intensity laser light, rather than high-power thermal lasers, could have beneficial medical applications, leading to the emergence of low-level laser therapy (LLLT).

### 1.3. Low-Level Light Therapy: Laser or LED—Which Is Better?

Early studies observed that light could either stimulate biological processes (healing) or inhibit them (inflammation), producing therapeutic effects in both directions. “Stimulation”, often used in the scientific literature, refers to positive or activating effects. The term “low-level” was considered vague, due to a lack of clear dosimetry and unified experimental protocols [11]. It was later shown that coherent laser light is not strictly necessary, as non-coherent light-emitting diodes (LEDs) can also be effective [12]. Two main advantages traditionally attributed to lasers are coherence and tissue penetration:

1. Light coherence.

The debate regarding the distinction between coherent and incoherent light lasted for decades. The stimulatory effects of light were first reported following the introduction of lasers as coherent light sources, particularly the He–Ne laser. In the early stages of research, coherence was considered a key factor responsible for the observed biological effects [13]. The subsequent introduction and widespread use of light-emitting diodes (LEDs), capable of emitting non-coherent light, helped clarify this assumption. Experimental evidence has progressively demonstrated that coherence was not responsible for therapeutic effects, as this property is lost within the superficial layers of biological tissues. In the absence of coherence, wavelength has been identified as the fundamental parameter governing biological effects [14,15].

2. Tissue penetration.

Penetration is mainly determined by the parameters of light (wavelength, irradiance, fluence, emission modality), as well as by the duration and frequency of exposure. The effects of light exposure follow a biphasic dose–response curve, meaning that an imbalance in these factors may produce the opposite outcome [16]. In addition, the scattering properties of the target tissue and the thickness of the barriers through which light must pass significantly influence penetration [17]. Red and near-infrared light penetrate more deeply than blue or green light, with penetration depths ranging from approximately 1 mm to 2–3 cm depending on wavelength (150–1200 nm) [18]. Both light sources can produce comparable therapeutic effects under appropriate parameters. However, unlike lasers, LEDs enable irradiation of larger tissue areas [19].

### 1.4. Conceptual Definition of Photobiomodulation (PBM)

LEDs can emit light that is close to a single wavelength, although their emission spectrum spans a broader range than that of lasers. Since experimental evidence suggests that non-coherent and quasi-monochromatic light, such as that emitted by LEDs, can be effective for low-level light therapy, it is reasonable to assume that natural broadband light may also produce similar biological effects [20]. Based on these considerations, the term “low-level laser therapy” was considered no longer appropriate, as this application also employed other types of light sources. This highlighted the lack of consistency and consensus in terminology. Consequently, a common agreement emerged on the use of the term photobiomodulation (PBM) to describe a form of light therapy utilizing non-ionizing light sources, including lasers, LEDs, and broadband light in the visible and infrared spectrum. PBM is a non-thermal process that involves endogenous chromophores (i.e., naturally present in the body), triggering photophysical (linear and nonlinear) and photochemical events across various biological scales [11]. This results in beneficial therapeutic outcomes including pain and inflammation reduction, modulation of the immune system, and promotion of wound healing and tissue regeneration.

### 1.5. The Modern Era and the Emergence of New Light Sources: From OLED to QLED

Organic light-emitting diodes (OLEDs):

Following LEDs, the emergence of organic light-emitting diodes (OLEDs) promised a revolution in medical applications. The new properties of this technology compared with previous generations offered innovative potential. OLEDs are flexible, wearable, provide uniform illumination over large areas, and have reduced form factor and device thickness. They were first proposed in photomedicine in 2009 [21,22]. Over approximately six years, additional OLED-based photomedical studies were published [23,24]. In 2019, significant improvements in device performance compared with early OLED systems were reported [25,26]. In particular, significant advances were achieved in emission bandwidth and wavelength tunability through the microcavity effect, which was originally introduced to enhance light extraction efficiency [18]. However, the main limitation of OLED technology remained its relatively broad emission spectrum and the power densities produced, which are typically lower than those required for standard photomedical applications.

Quantum Dot Light-Emitting Diodes (QLEDs):

It was only with the advent of quantum dot light-emitting diodes (QLEDs) that these limitations were overcome, as they are able to produce higher power outputs with a narrower emission spectrum. In these light sources, wavelength tunability is achieved directly through the synthesis of quantum dots (QDs), by optimizing the core–shell structure and charge injection, without requiring additional device design strategies [18]. In addition, they exhibit high fluorescence in the red region, which may enable improved performance in photomedical applications. A notable example is the inverted red QLED fabricated on glass with ultrahigh brightness reported in 2015, which represents the fundamental architecture of devices later used in photomedical studies [27]. In 2017, QLEDs were proposed for the first time as light sources in photodynamic therapy (PDT) and photobiomodulation (PBM) [28]. More recently, flexible QLEDs (FQLEDs) have also been developed, employing structures similar to hybrid QLED devices on glass but fabricated on flexible and biocompatible substrates.

The practical application of photomedicine suggests that both OLED and QLED light sources produce therapeutic effects comparable to those obtained with conventional light sources. Their relatively low production costs and scalable fabrication further promote their adoption as viable alternatives for outpatient applications. Flexibility, closely associated with ergonomics and wearability, together with easier access to therapy, represent two key innovative factors underlying their importance in modern clinical practice. In this context, these emerging light sources have paved the way for a significant evolution in treatment protocols, which are no longer confined exclusively to healthcare facilities but can also be extended to home-based settings. This transition could reduce healthcare costs by decreasing the need for specialist consultations and hospital-based treatments.

### 1.6. Contemporary Photomedicine and Future Perspectives

Current photomedicine research focuses on several areas, including the study and management of adverse effects of light exposure, the treatment of acute and chronic disorders, as well as applications in diagnosis, prevention, and treatment monitoring. Over time, therapeutic goals have evolved toward a deeper understanding of the biological mechanisms underlying light–tissue interactions. In parallel, technological progress has adapted to these needs, aiming to improve both accessibility to care and treatment efficacy. Consequently, the development of wearable and flexible devices with long operational lifetimes has assumed a central role in modern photomedical applications, including photodynamic therapy (PDT), photothermal therapy (PTT), and photobiomodulation (PBM). In particular, PDT is based on the interaction between a photosensitizing agent, light of a specific wavelength, and molecular oxygen, leading to the generation of reactive oxygen species that induce targeted cellular damage. Unlike PBM, which primarily modulates biological processes through non-thermal mechanisms, PDT is typically used for cytotoxic purposes, particularly in oncology and dermatology. PTT, instead, relies on the conversion of light energy into heat, inducing localized hyperthermia and temperature-dependent therapeutic effects ranging from reversible cellular stress to irreversible tissue damage. Currently, two main development directions have been identified for wearable photomedical devices intended for healthcare applications. The first concerns the integration of display and input/output systems, closely related to monitoring (e.g., displays sensitive to pressure or temperature variations, optical biosensors, theranostic systems, and related technologies) [29]. The second focuses on the development of flexible, large-area light sources capable of delivering controlled and uniform irradiation [30].

### 1.7. Research Questions and Analytical Framework

Although photobiomodulation (PBM) and light-based therapies have been extensively investigated from both clinical and technological perspectives, limited attention has been devoted to comprehensive bibliometric analyses that systematically trace the evolution of this research field over time. In particular, the rapid emergence of wearable photonic devices, especially patch-based systems, lacks clear contextualization within the broader historical and technological development of PBM research. This gap highlights the need for a structured bibliometric investigation that can identify publication trends, thematic evolution, and emerging research directions. In this context, tracing the historical evolution from first-generation laser systems to more recent nanotechnologies based on QDLEDs allows for a deeper understanding of how photomedical light sources have progressed through distinct phases, characterized by improvements in performance, spectral control, efficiency, and adaptability to wearable systems. These developments reflect an expanding and increasingly complex research landscape, supported by growing experimental, preclinical, and clinical evidence. Given the emerging and interdisciplinary nature of this field, the present study addresses three main research questions:

(1) What is the temporal and quantitative evolution of scientific production on light sources in photomedicine, and how has the transition toward wearable medical devices developed, with particular reference to photonic patches?
(2) Which thematic clusters and lexical co-occurrence patterns can be identified between traditional and modern light sources and wearable medical devices during the period 1970–2025?
(3) What thematic trajectories have guided the application of wearable photonic devices, including those based on nanotechnology and quantum dots, in the fields of balance, pain, and neuromodulation?

This study (Part I Section 1) represents the introductory component of a broader research project. Further analyses on photonic patches, including their technological features, application protocols, and clinical outcomes, will be addressed in Section 2, with specific focus on nanotechnology-based photonic patch systems, including Taopatch^®^ as a representative case study of quantum dot (QD)-based technologies. The contribution of these systems to therapeutic outcomes remains largely unquantified, highlighting the need for further rigorous investigations to determine their clinical effectiveness and reproducibility. Accordingly, this bibliometric analysis provides a framework to position phototherapeutic patch technologies within the broader landscape reported in the literature.

## 2. Materials and Methods

A comprehensive literature search (1970–2025) was conducted using three major bibliographic databases: Scopus, PubMed, and Web of Science. Search strings were adapted to the specific syntax of each database and included the following keywords: (“Taopatch” OR “photobiomodulation patch” OR “wearable light therapy patch” OR “wearable light-emitting patch” OR “wearable phototherapy device” OR “nanotechnology patch” OR “quantum dot patch”) AND (“postural control” OR “balance” OR “proprioception” OR “pain” OR “chronic pain” OR “mechanism of action” OR “biological effect” OR “muscular development” OR “neuromodulation”). Although the overall scope of the study addresses photobiomodulation and light-based therapies broadly, the search strategy was intentionally focused on wearable and patch-based technologies as a representative and rapidly evolving subset of this field. The search yielded 117 records distributed as follows across the databases: PubMed (59 records), Scopus (56 records), and Web of Science (2 records). No exclusion filters were applied prior to data processing, and the bibliometric analysis was conducted using the Bibliometrix R package (R software, version 4.5.0; R Foundation for Statistical Computing, Vienna, Austria) on the complete dataset [31]. Two records were excluded automatically due to missing metadata, and five duplicates or incomplete records were removed, resulting in a final dataset of 110 articles. No formal quality assessment or risk-of-bias evaluation was performed, and PRISMA guidelines were not applied due to the bibliometric design of the study. The analysis was conducted according to the TALL model (Tracking, Analysis, Layout, Learning).

### 2.1. Temporal Interval of Analysis

A longitudinal bibliometric framework (1970–2025) was adopted to capture the full technological and conceptual trajectory of light-based applications in photomedicine. The temporal segmentation (1970–2009; 2010–2023; 2024–2025) was theoretically guided by key technological turning points relevant to the field’s evolution.

- 1970–2009: Exploratory and early consolidation phase of low-level light therapy based on conventional sources (laser and LED), including initial experimental applications of OLED technologies.
- 2010–2023: Diffusion and consolidation of OLED-based platforms and maturation of quantum dot technologies, marking an expansion and technological legitimization phase.
- 2024–2025: The most recent stage, isolated to detect emerging research trends, with increasing thematic specialization and orientation toward clinical validation and translational integration.

The unequal duration of the intervals reflects the nonlinear development of the field: a prolonged formative phase, followed by accelerated technological expansion, and a final short interval designed to capture the most recent dynamics without temporal dilution. Given the moderate size of the dataset (110 documents), temporal segmentation required aggregation of periods to ensure statistical stability of the thematic evolution analysis. Initially, the second interval had been defined up to 2017, corresponding to the first application of QDLEDs in photomedicine, similar to the 2009 cutoff used for OLEDs. However, due to the lack of stable thematic structures under that segmentation, the software was unable to generate the analysis. Therefore, to ensure both methodological stability and consistency with a structurally significant event in the field’s development, the second temporal interval was definitively established in 2023, representing a decisive point marked by the global recognition of QDs through the Nobel Prize award [32].

### 2.2. Bibliometrix Parameters

Among the analysis parameters, the default settings provided by the Bibliometrix package were retained without modification. Specifically, the minimum cluster frequency was set to 5 (per 1000 documents), the number of words to 250, the minimum weight index to 0.1, and the label threshold to 0.3. Three labels were assigned to each thematic cluster using the Walktrap clustering algorithm. The Inclusion Index weighted by word occurrences was adopted to analyze thematic evolution. Temporal transitions and thematic stability across periods were assessed through the Weighted Inclusion Index (WI), Inclusion Index (II), Occurrences (O), and Stability Index (SI). WI and II (range 0–1) measure thematic relevance and conceptual overlap, respectively. Occurrences indicate the number of documents associated with each theme, while SI (range 0–1) reflects thematic persistence across periods [33]. Given the limited availability of Author Keywords in earlier periods, network and thematic analyses were conducted using Keywords Plus to ensure temporal consistency and longitudinal comparability.

## 3. Results

The analyses presented in this study should be interpreted within a bibliometric framework aimed at identifying scientific research patterns, rather than as a reconstruction of the technological evolution of light-emitting devices.

### 3.1. Tree-Map Analysis of the Main Areas of Research

**Figure 1:**
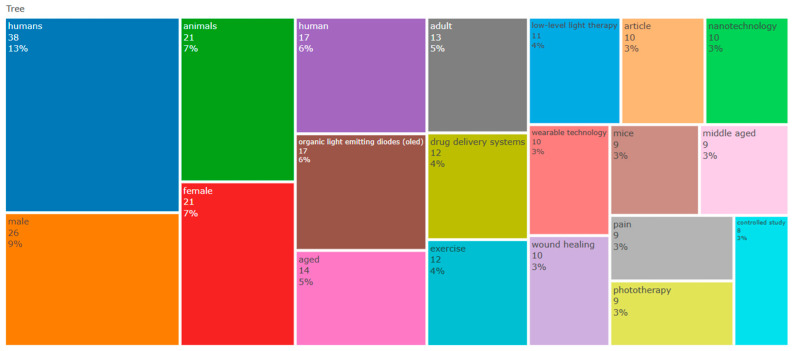
Tree Map—Thematic structure of the literature (1970- 2025). Each category is represented by a proportional block indicating the number of articles and their relative percentage within the overall dataset (only the 20 most frequent topics were visualized; percentages may differ from the total dataset).

The tree-map analysis revealed the main research topics within the dataset. The most frequent thematic categories were “humans” (8%), “male” (6%), “female” (5%), and “animals” (5%). Technology-related topics included “organic light-emitting diodes (OLED)” (4%), “drug delivery systems” (3%), “wearable technology” (2%), and “nanotechnology” (2%), with “smart textiles” and “electronic devices” appearing at a lower frequency (1%). Clinically oriented topics comprised “wound healing,” “pain,” “phototherapy,” “photobiomodulation,” “aging,” and “quality of life” (2–3%). Additional categories identified in the dataset included “COVID-19,” “controlled study,” and “treatment outcome” (Figure 1).

### 3.2. Annual Scientific Production and Average Citations per Year

**Figure 2:**
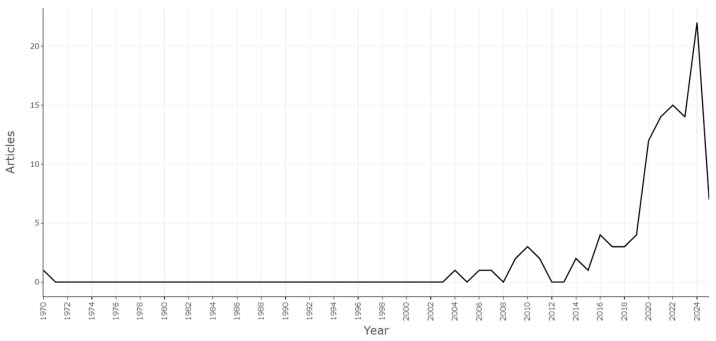
Annual scientific production (1970–2025). The x-axis represents the publication years, while the y-axis indicates the number of documents produced each year.

The analysis of annual scientific production revealed a highly asymmetric temporal trend. From the 1970s to the early 2000s, research output remained extremely limited, with fewer than 1–2 publications per year. This minimal level of scientific output reflects the lack of a structured research framework at that time, consistent with the subsequent emergence and consolidation of the sector observed in the decades that followed. A gradual increase was observed around 2010, followed by sustained growth beginning in 2019. The peak of publications occurred in 2024, with over 20 articles published, indicating a very recent and concentrated increase in research activity (Figure 2). The analysis of average citations per year shows a similar trend, with values close to zero until 2015, followed by a sharp increase between 2020 and 2022 (over 15 citations per year).

### 3.3. Bradford’s Law and Lotka’s Law

**Figure 3:**
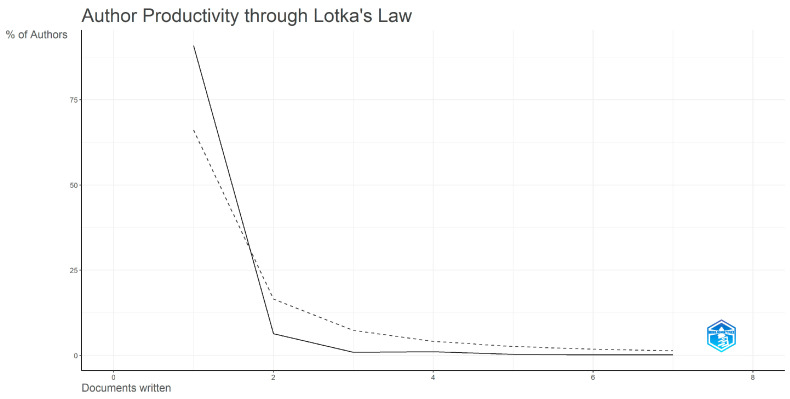
Lotka’s Law Author Productivity Distribution: The x-axis indicates the number of documents written and the y-axis the corresponding percentage of authors. The solid line shows the observed distribution, while the dashed line represents Lotka’s theoretical predictive distribution used for comparison.

The main journals belong to the areas of materials science, biotechnology, and nanomedicine. The analysis of sources using Bradford’s Law highlights a core group of journals (‘core sources’) that publish the majority of relevant articles. Among them, *Advanced Materials* and *International Journal of Environmental Research and Public Health* are the journals with the highest number of publications (Table 1). The distribution of author productivity was analyzed according to Lotka’s Law. The results show that most authors (approximately 75–80%) published only one article on the topic, while a smaller proportion (approximately 20%) published two articles, and a marginal percentage published three or more articles (Figure 3). Kim Y. emerged as the most prolific author (seven publications), followed by Kim H. and Jeong Y. (five and four publications, respectively). The observed distribution reflects a pattern typical of emerging research fields. At the institutional level, the highest scientific output was associated with the Biophotonics Applied to Health Sciences group at Nove de Julho University (nine articles), followed by Sungkyunkwan University and the Unit of Rehabilitation, Research Laboratory of Biomechanics and Rehabilitation (eight articles each).

### 3.4. Thematic Evolution in Titles, Keywords, and Abstracts (1970–2025)

**Figure 4:**
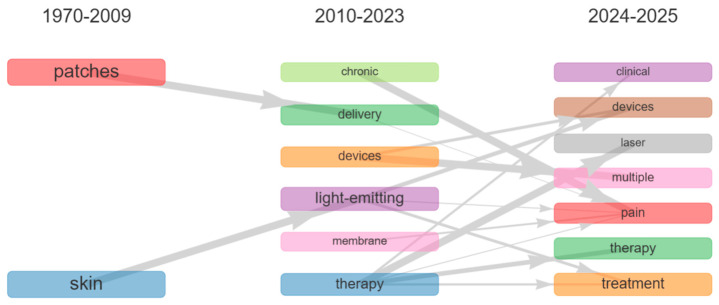
Thematic evolution map of the literature based on “titles” (1970–2025). The evolution of themes across time periods was computed using the Inclusion Index weighted by word occurrences. Line thickness is proportional to the Weighted Inclusion Index, indicating the degree of thematic continuity across clusters. A minimum weight threshold of 0.1 was applied to filter weak connections.

The analysis of article titles published between 1970 and 2025 highlights several relevant transformations (Figure 4). Historical terms such as “patches” and “skin,” present in the early period (1970–2009), gradually shifted in subsequent years toward “delivery” and “light-emitting” respectively. This transition suggests a differentiated progressive development: in the first case, toward patch-based delivery systems, and in the second, toward skin applications based on light. In the 2010–2023 period, a thematic expansion and diversification can be observed, with growing interest in biomedical devices, therapeutic approaches, and management of chronic conditions. A further specialization emerges in the most recent period (2024–2025), where the term “light-emitting” predominantly converges toward “devices” (O = 8; WI = 1.0; II = 1.0; SI = 1.0), indicating a strong and stable relationship. Additionally, further connections, as observed for the term “therapy”, extend to “treatment” (O = 9; WI = 0.41; II = 0.11; SI = 0.04), “pain” (O = 5; WI = 0.10; II = 0.10; SI = 0.03), and “multiple” (O = 3; WI = 0.50; II = 0.50; SI = 0.05), showing weak but conceptually consistent relationships. The presence of the term “laser,” although referring to a traditional light source, signals a shift in research focus, consolidating the laser as a clinically validated therapeutic tool.

**Figure 5:**
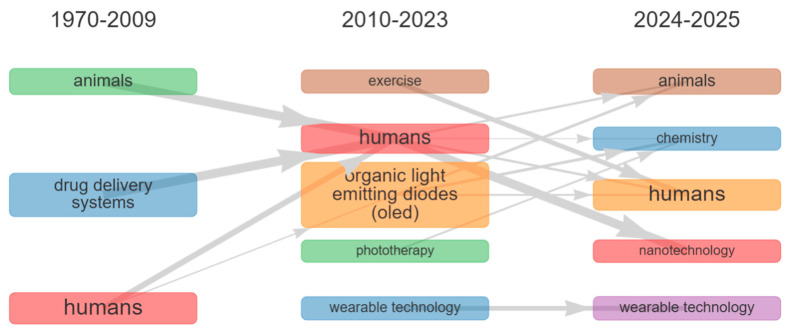
Thematic evolution map of the literature based on “keywords” (1970–2025). The evolution of themes across time periods was computed using the Inclusion Index weighted by word occurrences. Line thickness is proportional to the Weighted Inclusion Index, indicating the degree of thematic continuity between clusters. A minimum weight threshold of 0.1 was applied to filter weak connections.

The analysis of keywords reveals particularly relevant evolutionary steps (Figure 5). In the period 1970–2009, three main thematic clusters emerge: “animals,” “drug delivery systems,” and “humans.” During this phase, research appears focused on preclinical studies using animal models, the development of drug delivery systems, and early clinical applications. In the intermediate period (2010–2023), these terms converge primarily into “humans” (mean values: O = 2.6; WI = 0.89; II = 0.40; SI = 0.03), suggesting a strong and relatively continuous thematic transition, characteristic of the consolidation of human experimentation. This term represents the central node in this development phase, acting as a semantic hub around which terms such as “OLED,” “phototherapy,” and “wearable technology” are organized. Compared to the previous period, the thematic structure expands from purely pharmacological administration to advanced light-based technologies, phototherapeutic applications, and wearable devices. In the most recent period (2024–2025), a semantic reorganization is observed. The convergences of “humans” (O = 12; WI = 0.26; II = 0.08; SI = 0.02) and “OLED” (O = 4; WI = 0.32; II = 0.08; SI = 0.02) toward “animals” suggest connections related to a renewed interest in preclinical studies following new formulations or innovative materials. Further connections with the terms “chemistry” (O = 8; WI = 0.11; II = 0.11; SI = 0.03) and “nanotechnology” (O = 6; WI = 1.0; II = 0.50; SI = 0.03) confirm a strengthening of the material and engineering dimension in this phase. Transitions for “chemistry” appear weak and emerging, whereas those for “nanotechnology” are strong and more consolidating. The continuity of the “wearable technology” node between the intermediate and final periods signals maturation and stabilization of the field.

**Figure 6:**
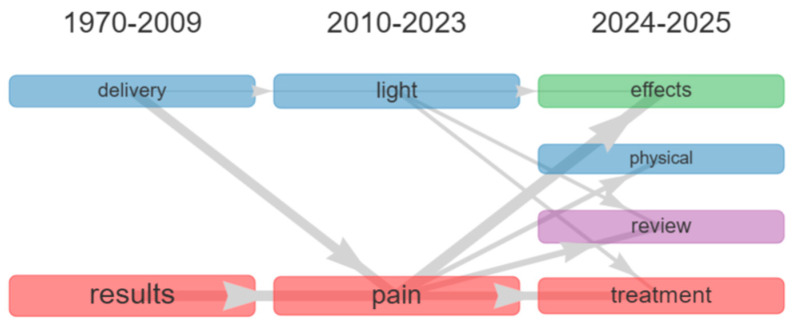
Thematic evolution map of the literature based on “abstracts” (1970–2025). The evolution of themes across time periods was computed using the Inclusion Index weighted by word occurrences. Line thickness is proportional to the Weighted Inclusion Index, indicating the degree of thematic continuity between clusters. A minimum weight threshold of 0.1 was applied to filter weak connections.

The analysis of abstracts shows that terms such as “delivery” (O = 2; WI = 0.50; II = 0.13; SI = 0.01) and “results” (O = 2; WI = 0.55; II = 0.04; SI = 0.01), present in the early research period (1970–2009), both converge in the intermediate period (2010–2023) toward “pain”, albeit with low frequencies and emerging connections (Figure 6). The semantic bridge between the time intervals is represented by the terms delivery, improve, patches, patients, treatment, application, skin, identifying the conceptual link between these phases. In the most recent period (2024–2025), a relatively strong association is observed between the term “pain” and “effects” (O = 17; WI = 0.59; II = 0.03; SI = 0.01), which appears as a highly connected theme. Moreover, the connection of the term “light” with “review” (O = 15; WI = 0.24; II = 0.02; SI = 0.01) and “treatment” (O = 25; WI = 0.25; II = 0.01; SI = 0.01), although moderate, suggests the presence of review-oriented publications, along with a increasing references to clinical applications. The semantic bridge connecting the intermediate and final periods is represented by the terms wearable, healthcare, medical, photonic, textiles, monitoring, application, light-emitting, OLED, LED, photobiomodulation, parameters, quantum, and implantable, which may be interpreted as indicators of emerging research trends.

### 3.5. Thematic Map: Titles, Keywords and Abstracts (1970–2025)

**Figure 7:**
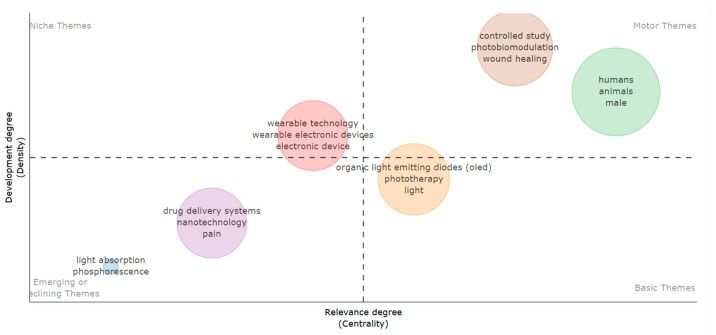
Thematic map (1970–2025). The upper-right quadrant identifies motor themes, the lower-right quadrant basic themes, the upper-left niche themes, and the lower-left emerging or declining themes.

Thematic analysis highlights the conceptual structure of the research field (Figure 7). Among the motor themes, central and well-established topics (photobiomodulation, wound healing, and studies on humans and animals) are identified. These themes represent the driving core of the research, indicative of high internal cohesion and strong connections with other thematic areas. Basic themes (light, phototherapy, organic light-emitting diodes—OLED) are configured as fundamental concepts, broadly connected but still requiring further exploration. Conversely, niche themes (wearable technology, wearable electronic devices, electronic device) show a high degree of development but lower centrality, suggesting more specific and specialized areas, potentially oriented toward emerging technological applications. Finally, emerging or declining themes (drug delivery systems, nanotechnology, pain, light absorption, phosphorescence) indicate less developed or marginal areas, which may represent early-stage research lines or sectors gradually decreasing in interest. Overall, the thematic map reflects a research field in evolution, in which photobiomodulation and tissue healing constitute the main axes, while integration with wearable technologies and nanotechnologies suggests new interdisciplinary development directions.

### 3.6. Keyword Trends in the Scientific Literature (1970–2025)

**Figure 8:**
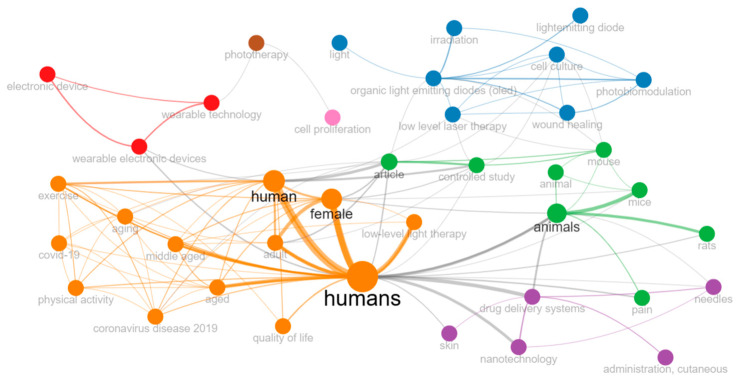
Co-occurrence network of recurrent “keywords” in the scientific literature (1970–2025). Thicker and darker lines indicate stronger co-occurrence links, while thinner and lighter lines reflect weaker associations; similarly, larger bubbles represent more frequent or influential keywords.

Keyword trends highlight a significant temporal evolution of research topics (Figure 8). Terms such as “low-level light therapy” (frequency = 11; median = 2018; IQR = 2010–2022) and “nanotechnology” (frequency = 10; median = 2017; IQR = 2014–2018) show increased presence in recent years, with initial development in the early 2010s. Between 2020 and 2021, a translational shift emerges with a substantial increase in the occurrence of specific concepts. Keywords related to technological development and clinical integration, including “phototherapy” (frequency = 9; median = 2020; IQR = 2019–2021), “wearable electronic device” (frequency = 8; median = 2020; IQR = 2020–2022), “OLED” (frequency = 17; median = 2021; IQR = 2020–2023), and “wound healing” (frequency = 10; median = 2021; IQR = 2021–2024), reach their peak median frequencies in these years. The term “OLED” reflects the temporal gap between its initial technological development and its subsequent recognition as a clinically relevant light source. The high frequency and wide temporal distribution of the term “humans” (frequency = 38; median = 2021; IQR = 2016–2024) indicate a shift toward population-based research. More recent keywords peak between 2023 and 2024, including “treatment” (frequency = 14; median = 2023; IQR = 2017–2024), “exercise” (frequency = 12; median = 2023; IQR = 2021–2024), “aging” (frequency = 7; median = 2024; IQR = 2024–2024), and “physical activity” (frequency = 7; median = 2024; IQR = 2022–2024). These trends suggest an expansion toward multimodal therapeutic approaches and broader public health applications. Finally, “application prospect” and “biosensing” (frequency = 1; median = 2025; IQR = 2025–2025) indicate emerging directions focused on personalized monitoring and future translational applications.

## 4. Discussion

### 4.1. Research Question 1: Temporal Evolution and Transition Toward Wearable Devices

The conducted analysis indicates that this research field was, at least until a few years ago, relatively niche and fragmented. Scientific production remained limited for a long time, with a low number of annual publications and similarly low visibility. Significant growth was observed only from 2019 onwards, culminating in a peak of publications in 2024, suggesting the emergence of collective interest in more advanced therapeutic and technological photonic solutions. Early research directions on patches for light-based therapies were driven by the development of the first drug delivery and light-emitting systems, primarily oriented toward preclinical investigations and cutaneous applications. The transitional period, between 2010 and 2023, represents a phase of convergence for research in this field. With the introduction of new technological innovations, especially OLED technology, a broader scientific and thematic landscape emerged, opening significant development pathways in phototherapy and wearable devices. This evolution reflects the transition from traditional laser systems, often bulky and stationary, toward flexible, lightweight, and wearable solutions. Human experimentation has progressively consolidated since 2016 and continues to expand. Nanotechnology, associated with light-based therapies, has become more prominent in the most recent period, although it appeared as a research theme in this field as early as 2014. Despite technological advancements, lasers remain a light source widely used in photomedicine, probably due to their mature and well-established technological development. Wearable devices based on innovative light sources are still largely in the preclinical phase, with research objectives mainly focused on evaluating therapeutic effects in pain management and chronic diseases. Recent research increasingly addresses advanced application prospects and biosensing systems. Moreover, the advent of the IoT era and the possibility of managing adaptive and autonomous treatments through everyday smart devices have enabled the integration of photonic patches into networks, allowing remote monitoring capabilities. These trends appear to project future developments toward the paradigm of telemedicine.

### 4.2. Research Question 2: Traditional and Modern Light Sources and Wearable Medical Devices

Among modern light sources, OLED technology is the most representative. However, the semantic transition toward the most recent research period highlights associations with quantum mechanisms and implantable technologies. This observation is supported by the literature, which emphasizes the advanced potential of bioelectronic components for light-based diagnosis and therapy. Advancements in optoelectronics and materials science have improved the optical interface with biological tissues, enabling a wide range of device configurations, including pills, injectable probes, and miniaturized implants, mainly composed of light-responsive biomaterials [34]. Secondly, the literature recognizes the use of electronic textiles such as smart clothing (t-shirts, shoes) and accessories (bracelets, glasses, watches) in photomedicine [35]. These systems may incorporate properties related to light emission, heat transfer or storage, and complex sensing capabilities to support health monitoring and disease prevention [36,37,38]. Currently, wearable photonic patches are considered to be among the most promising platforms in photomedicine [39]. In most cases, their light emission is based on OLED and LED sources, primarily applied in photodynamic therapy [22,40]. Additional evidence supports their use in photothermal therapy, with wireless operation and remote control via smartphones [41]. Other systems are applied in photobiomodulation for rehabilitation purposes [42,43]. QD-based patches remain limited, consistent with the early-stage research phase of this specific technological interface. Preclinical evidence demonstrates their potential related to emission spectrum control and physiological monitoring, properties of fundamental importance for modern clinical applications [44]. Table 2 summarizes the key performance characteristics and application-related features of the main photomedical light technologies.

### 4.3. Research Question 3: Application Trajectories and Future Perspectives

The observed lexical transformation is not random, but reflects the structured evolution of the field, indicating a structural reconfiguration of the research domain: from an initially theoretical or preclinical phase to an applied, patient-centered approach focused on the clinical effectiveness of proposed solutions. Overall, the literature is undergoing a phase of critical reassessment aimed at achieving a deeper understanding of the topic, which may provide a foundation for future advancements. “Pain” is among the most recurrent terms in 2010–2023, representing an application domain of growing interest in this phase. This trend has persisted in the most recent research periods (2024–2025). In contrast, balance and neuromodulation do not emerge as significant thematic nodes in the application of light-based therapies. For photonic patches, beyond the oncological field, skin regeneration, wound healing, and hair loss represent the most common application areas to date [40,44,45,46]. The field is moving toward the concept of “tailored medicine”, increasingly integrated into people’s daily lives, thanks to portable, lightweight, and intelligent technologies. From a structural perspective of the scientific field, results confirm a configuration typical of emerging sectors (mean documents per year = 6.05; annual growth rate = +3.6%). This is a rapidly developing field, still establishing its theoretical and methodological framework. In summary, the analysis portrays a sector in full transformation, driven by technological innovation but increasingly oriented toward concrete clinical application. Future prospects point toward closer integration among biotechnology, materials science and personalized medicine, with growing attention to patient quality of life and rigorous evaluation of therapeutic outcomes. If these trends are confirmed, the field is likely to continue growing in the coming years, both in terms of scientific production and tangible impact on clinical practice.

### 4.4. Is the Field Truly Reaching Clinical Maturity or Still in an Experimental Technological Phase?

One of the main critical issues characterizing wearable photonic devices, similar to observations in the broader field of health-related biotechnology and nanomedicine, is the persistent gap between laboratory research and clinical application. In particular, significant difficulties remain in translating innovations developed in experimental settings into clinically validated and commercially sustainable products capable of generating tangible benefits for patients [47,48]. The bench-to-bedside paradigm, embedded within the translational research model, describes a multidisciplinary process that extends from basic scientific discovery to the development of diagnostic tools, therapeutic protocols, and healthcare delivery strategies [49,50]. Within this interpretative framework, wearable photonic patches appear to be positioned at an intermediate translational stage. On one hand, technological maturity, especially in materials science and optoelectronics, appears significantly advanced. On the other hand, clinical validation remains limited and characterized by considerable methodological heterogeneity (e.g., sample size, light sources, intervention objectives, therapeutic modalities, etc.). From this perspective, future progress will depend not only on continued technological development but also on the establishment of shared dosimetric standards and the conduct of methodologically robust clinical trials. Bridging this translational gap will be a decisive factor in determining whether these technologies evolve into standardized therapeutic platforms integrated into clinical practice or remain promising yet predominantly experimental solutions. Future studies should incorporate patent analyses to provide complementary insights into technological development and innovation transfer in photonic technologies.

## 5. Conclusions

Overall, the quantitative analysis highlights an expanding scientific field characterized by rapid evolution both terminologically and conceptually. In photomedicine, particularly photobiomodulation (PBM), the emergence of new wearable technologies coincides with increasing interest in clinical application, treatment personalization, and outcome assessment. This transformation suggests that research is moving beyond the exploration of innovative solutions, increasingly aiming to understand their real impact on health and quality of life. In addition to mapping scientific production, this study provides a structural interpretation of the technological transition in photomedicine, identifying developmental phases and emerging application trajectories. These insights may guide future research priorities and funding strategies.

## Figures and Tables

**Table 1:** Top 5 journals according to Bradford’s Law. Journals are ranked in descending order of publication frequency and cumulative frequency. The table highlights the core sources contributing to the majority of articles in the dataset.

Journals	Rank	Freq	CumFreq
*Advanced Materials*	1	6	6
*International Journal of Environmental Research and Public Health*	2	4	10
*Acs Applied Materials and Interfaces*	3	2	12
*Acs Nano*	4	2	14
*Drug Delivery and Translational Research*	5	2	16

**Table 2:** Comparasion of the main light-based technologies used in photomedicine in terms of physical properties, clinical maturity, and degree of integration. Laser systems are clinically established but are predominantly stationary due to low flexibility. LEDs are widely used and offer moderate portability. OLEDs are emerging technologies characterized by high flexibility and suitability for wearable applications. QLEDs represent a promising early-stage platform with enhanced spectral control and potential for future wearable and bio-integrated implementation.

Technology	Output Power	Flexibility	Spectral Control	Photomedical Use	Wearability/ Integration
**LASER**	Very high	Low	Extremely high	Clinically established	Stationary systems
**LED**	Moderate–high	Moderate	Moderate	Widely used	Portable systems
**OLED**	Low–moderate	Very high	Low	Emerging	Wearable systems
**QLED**	Moderate–high	High	Very high	Promising/ early-stage	Emerging bio-integrated systems

## Data Availability

Data used in this study are derived from publicly available scientific literature and bibliographic databases.

## Abbreviations

The following abbreviations are used in this manuscript:

PBM	Photobiomodulation
LLLT	Low-level laser therapy
LEDs	Light-emitting diodes
OLEDs	Organic light-emitting diodes
QDLEDs	Quantum dot light-emitting diodes
PDT	Photodynamic therapy
PTT	Photothermal therapy
IoT	Internet of Things
